# A Comparison of Five Animal Models for Acute Intervertebral Disc Herniation Research

**DOI:** 10.1002/jsp2.70116

**Published:** 2025-09-24

**Authors:** Thomas D. Slater, Beatrice Gagliostri, Matthew J. Kibble, Nazli S. Tümer, Peter A. Cripton, Nicolas Newell

**Affiliations:** ^1^ Department of Bioengineering, Imperial College London London UK; ^2^ Department of Biomechanical Engineering, Faculty of Mechanical Engineering Delft University of Technology (TU Delft) Delft the Netherlands; ^3^ School of Biomedical Engineering University of British Columbia Vancouver Canada

**Keywords:** bovine lumbar discs, bovine tail discs, flexion, mechanism of herniation, microstructural analysis, ovine lumbar discs, porcine cervical discs, porcine lumbar discs, standardized loading protocol, ultimate compression

## Abstract

**Study Design:**

Microstructural investigation of mechanical load induced acute disc herniation on five animal models.

**Objective:**

To compare how spinal discs in different animal models herniate under a standardized complex compressive load.

**Summary of Background Data:**

Animal models in disc herniation research offer reduced degeneration‐associated variability, lower cost, and greater availability compared to human specimens. However, there is limited consensus regarding which species is best suited for modeling human herniation, making a comprehensive comparison of species‐specific herniation mechanisms necessary.

**Materials and Methods:**

A standardized shear and compressive load, designed to herniate intervertebral discs, was applied to isolated discs of five cadaveric animal models (*n* = 30, 6 specimens per group): bovine tail, bovine lumbar, ovine lumbar, porcine lumbar, and porcine cervical. The segments were flexed (7°), and a shear‐compressive load was applied at a crosshead displacement rate of 40 mm min^−1^, until a force drop, or a displacement limit was reached (~80% of disc height). Microstructural analysis was undertaken to identify failure modes.

**Results:**

Clinically relevant herniation features were observed in all models—including endplate and annulus fibrosus (AF) tearing, AF delamination, vertebral body (VB) fracture, nucleus pulposus (NP) extrusion into VB, and radial NP movement. Bovine lumbar, porcine cervical, and porcine lumbar segments exhibited high rates of radial NP movement (84%, 100%, and 67%, respectively), with ovine lumbar discs displaying VB fracture (84%) and NP extrusions into the VB (67%). Bovine tail discs showed minimal damage but were characterized by sequential lamellar AF tears (67%).

**Conclusions:**

Porcine cervical, bovine lumbar, and porcine lumbar discs are suitable for annulus‐failure herniation research, although porcine cervical discs may be the most appropriate due to exhibiting the highest rate of relevant damages. Ovine lumbar discs are relevant for studying endplate junction failure herniations, and bovine tail discs are appropriate for implant‐related studies.

## Graphical abstract

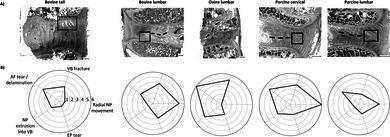

## 1 Introduction

Lower back pain is considered the primary contributor to the global non‐fatal health burden [1], with lumbar disc herniation being implicated in 3%–5% of cases [2, 3]. Acute disc herniation occurs when the nucleus pulposus (NP) or annulus fibrosus (AF) has protruded or extruded outside of its original confines [4], sometimes as a result of high mechanical load or sudden injury [5]. The protruded tissue can compress nearby nerves and contribute to inflammation within the spinal canal [4, 6], which often results in lower back pain, leg pain, or numbness (sciatica) [7]. Understanding the biomechanical causes and mechanisms of acute herniation is critical for improving interventions and treatments, aiming to prevent acute injuries, mitigate suffering, reduce sick leave, and enhance quality of life.

Biomechanical studies often use cadaveric human or animal tissue to recreate herniation ex vivo [8]. Animal tissue is frequently chosen for spinal disc research because it provides greater consistency than human tissue, exhibiting less variability in anatomical and pathological conditions, with minimal disc degeneration [8]. Animal models have been successfully used to infer the complete mechanisms of herniation, demonstrating that it can occur either via failure of the mid‐AF tissue or through failure of the endplate and vertebra, leading to an endplate junction failure (EPJF) [9, 10]. These findings align with clinical observations of herniation [11, 12].

The mechanism that is inferred is partially dependent on the loading type applied and any initial defects present in the sample. For example, cyclic loading tests lead to a more gradual failure of the disc than traumatic loading tests and may be seen as more physiological [13], but still produce both EPJF and AF‐failure herniations within the disc [14]. Under cyclic loading, the AF may progressively weaken and buckle, causing the NP to shift toward the posterolateral region, leading to disc failure in this region as the disc material becomes compacted [15]. Any initial defects present in the disc may also influence the pathway of failure [16, 17].

Old, degenerate discs rarely herniate symptomatically [3], suggesting that sufficient internal hydrostatic pressure is a key requirement for herniation. In contrast, supra‐hydrated discs may be prone to herniation due to elevated internal pressures, which increase annular stress, promote tearing, and ultimately lead to disc failure [18]. This phenomenon is evident in astronauts returning from space, where the absence of gravity leads to hyper‐swollen discs and an observed 80% rate of cervical disc herniation [19]; with similar mechanisms proposed for lumbar herniation [20]. These findings suggest that to mechanically induce herniation, a key objective is to elevate intradiscal hydrostatic pressure sufficiently to provoke structural failure within the disc.

The rate at which the spine is loaded is important due to the viscoelastic nature of the disc [21]. Higher strain rates increase disc stiffness [22], which alters internal strain patterns and may raise the likelihood of vertebral failure. This also contributes to more extensive radial tearing of the AF [10, 23].

Various animal species' discs have been used in herniation research [8, 24, 25]; including ovine lumbar [9, 10, 16, 23, 26, 27]; porcine lumbar [28, 29, 30, 31]; porcine cervical [32, 33, 34, 35]; bovine lumbar [36, 37]; and bovine tail discs [36, 38, 39]. Although these models have been compared anatomically [40, 41], biomechanically [8, 42, 43, 44], and biochemically [8, 45], no specific study has examined how each disc sustains microstructural damage under loads simulating herniation [8]. Consequently, it is unclear how the choice of animal model may affect the herniation mechanism inferred, and whether certain animal models are predisposed to certain modes of failure and what model should be selected for specific human disc herniation studies.

Therefore, we hypothesize that applying a moderately rapid compressive load to non‐degenerate discs, capable of generating sufficient hydrostatic pressure, will induce herniation. However, the mechanisms and specific microstructural failure patterns may vary due to anatomical differences between animal models.

This study aims to mechanically herniate discs with a standardized loading and identify and compare microstructural failure modes across animal models. The data will inform herniation mechanism inferences and provide insights into which models most accurately replicate human disc herniations, thus improving future animal model selection for herniation studies.

## 2 Materials and Methods

### 2.1 Sample Preparation

Thirty motion segments from five animal models (Table 1) were sourced from a local abattoir and subsequently frozen: bovine tail (*n* = 6), bovine lumbar (*n* = 6), ovine lumbar (*n* = 6), porcine lumbar (*n* = 6), and porcine cervical (*n* = 6). Suitable animal models and spine regions were chosen based on previous studies [31, 33, 40, 43]. Each specimen was dissected to remove all soft tissue and facet joints, leaving the intervertebral disc and anterior and posterior longitudinal ligaments (ALL and PLL) intact. The specimens were band‐sawed into vertebra‐disc‐vertebra segments, with fluoroscopy (Fluorescent InSightFD Mini C‐arm, Hologic, MA) confirming parallel endplates to the base. Three screws were inserted into each vertebra, and then the screws and half the vertebral body (VB) were encased in polymethyl methacrylate (PMMA) for attachment to the mechanical loading rig. Throughout the study, except during dissection and the complex‐compression application, segments were kept hydrated by wrapping in tissue soaked with 0.15 M phosphate‐buffered saline and by regularly spraying the specimens with the same solution. Freeze–thaw cycles were minimized throughout the testing protocol (3 times per motion segment) [46, 47].

**TABLE 1:** Detailed information on the discs used: by species, spinal region, vertebral levels.

Species	Number of specimens	Segment levels (number)
Bovine tail	3	Cd1‐Cd2 (3); Cd2‐Cd3 (3)
Bovine lumbar	3	L2‐L3 (3); L4‐L5 (3)
Ovine lumbar	2	L1‐L2 (2); L3‐L4 (2); L5‐L6 (2)
Porcine lumbar	3	L2‐L3 (3); L4‐L5 (3)
Porcine cervical	3	C3‐C4 (3); C5‐C6 (3)

*Note:* The discs tested from all species were skeletally immature, which was appropriate for the study's aim of modeling early‐stage mechanical injury in structurally intact tissue.

### 2.2 Geometric Measurements

Fluoroscopic images were obtained in both the sagittal and coronal planes before testing to measure disc width, depth, and height. In each plane, a digital reference line was drawn approximately parallel to both endplates at the disc's midpoint. The length of this line was calibrated using a 1 mm metal distance spacer placed in the same plane as the disc. Disc height was determined by measuring the perpendicular distance from the superior endplate to the inferior endplate at the center of the reference line. Measurements were performed by two researchers in ImageJ (v.1.54 g), with disc height being calculated as the average from both posterior and lateral views, and the disc area calculated assuming an elliptical shape. The apparent stress at failure was calculated by dividing the peak force by the area of the disc.

### 2.3 Mechanical Setup

**FIGURE 1:**
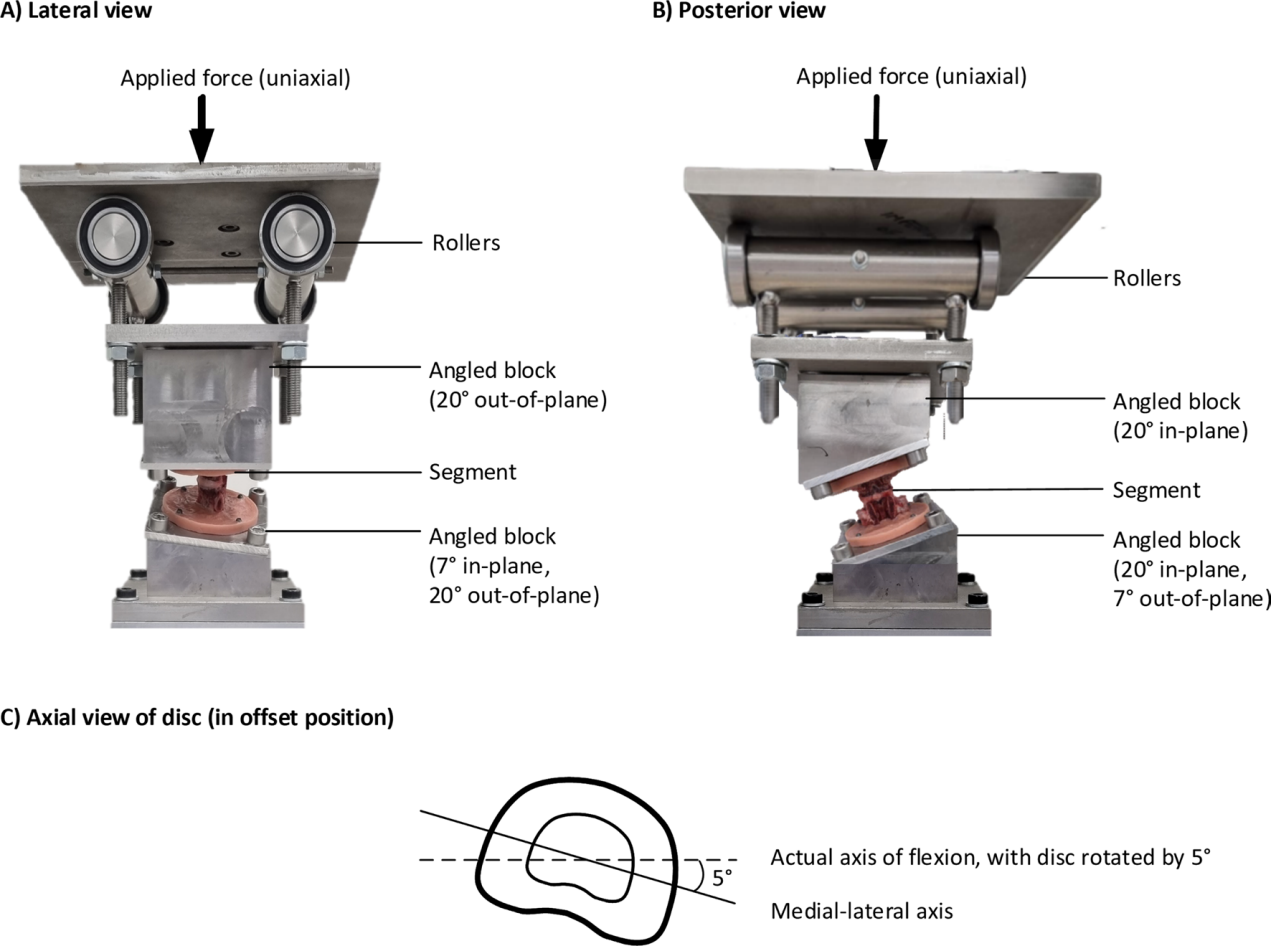
Rig configuration shown from a lateral (A) and a posterior view (B). The disc was flexed 7° (lateral view) and positioned with a lateral tilt of 20° (posterior view). The flexion and lateral tilt cause the vertical loading to apply both compressive, posterior shear, and left‐lateral shear forces to the disc. (C) Axial view of the disc, illustrating the actual axis of flexion, which is offset by 5° from the medial‐lateral axis. This offset results in a combined flexion and lateral bending, and also causes the applied shear force (which acts parallel to the actual axis of flexion) to include both lateral and posterior shear components.

A custom‐made complex‐posture fixture was compressed using a servo‐hydraulic materials testing machine (Model 8874, Instron, Norwood, MA). The fixture was designed to be mechanically similar to the rig used by Wade et al. (Figure 1) [48]. The setup allows the vertical cross‐head displacement to be resolved into posterior shear and left‐lateral shear force components, along with an axial force component while maintaining the disc in a constant flexed posture of 7°. The left‐lateral shear force is a result of the segment being compressed at a 20° angle when viewed in the frontal plane, whereas the posterior shear results from the 7° of flexion. The segment was also rotated 5° clockwise about the cranio‐caudal axis to introduce a left‐lateral bending component to the disc (Figure 1C). This posture was designed to strain the right‐paracentral region of the disc [49, 50], which is the area most commonly herniated in vivo [51]. This configuration mimics the spine during lifting activities involving asymmetric postures [23, 48].

### 2.4 Testing Protocol

A loading rate of 40 mm min^−1^ was selected because it has been shown to result in fewer vertebral fractures (33% vs. 53%) while still producing sufficient annular tearing (38% direct‐radial tears; 100% non‐continuous tears) [9, 23], when compared to load rates of 400 mm min^−1^, thereby balancing herniations caused by EPJF and AF failure. This moderate loading rate and short test duration (5–10 s) were chosen to further limit time‐dependent fluid loss [52], whilst still promoting internal fluid redistribution and the relevant damage mechanisms [48]. The compression was halted upon either a 15% load drop or if a maximum driven displacement was reached, to prevent further damage to the segment once failure had occurred [9]. The maximum displacement varied by animal model and was determined from preliminary tests that identified force‐drop failure points during disc compression. Limits were set ~25% above these points to allow for segment variability: 7 mm (bovine tail), 5 mm (bovine lumbar), 3 mm (ovine lumbar), 3 mm (porcine lumbar), and 3 mm (porcine cervical). These values corresponded to 80%–128% of disc height, depending on the failure susceptibility for each animal model. All segments were subjected to a nominal preload of 50 N for 15 min to allow the sample to come to equilibrium at the height change induced by the load. This low‐magnitude preload (~0.1 MPa) was intended to induce minimal fluid loss, as it remains well below typical in vivo quadrupedal lumbar intradiscal pressures during daily activities [53, 54], whilst still providing a consistent baseline prior to further loading.

### 2.5 Audio‐Visual Measurements

During testing, sagittal and lateral video (24 Hz) and audio (8 kHz) recordings were captured. Videos were reviewed for posterior‐lateral disc/vertebra displacement and fluid ejection. The audio was categorized as quiet, continuous cracks (noise present for 0.5+ seconds), or a single loud snap (< 0.5 s). Both categories were reviewed independently by two researchers (BG and TS), achieving 100% consensus.

### 2.6 Fixation and Sectioning

Following loading, the discs were fixed with 10% neutral‐buffered formalin for 1 week. The fixed discs were then decalcified in 10% formic acid for a further 2 weeks. The discs were sagittally bisected and serially sectioned with a cryotome (CryoStar NX70, Epredia, Portsmouth, NH) to obtain 50 μm sections that were at 45° to the sagittal plane. The sections were wet‐mounted and imaged in brightfield using an inverted microscope (MICA Microhub, Leica, Wetzlar, DE). All sections were inspected throughout to ensure slices where damage was most easily identifiable were chosen.

### 2.7 Microscopic Damage Examination

**FIGURE 2:**
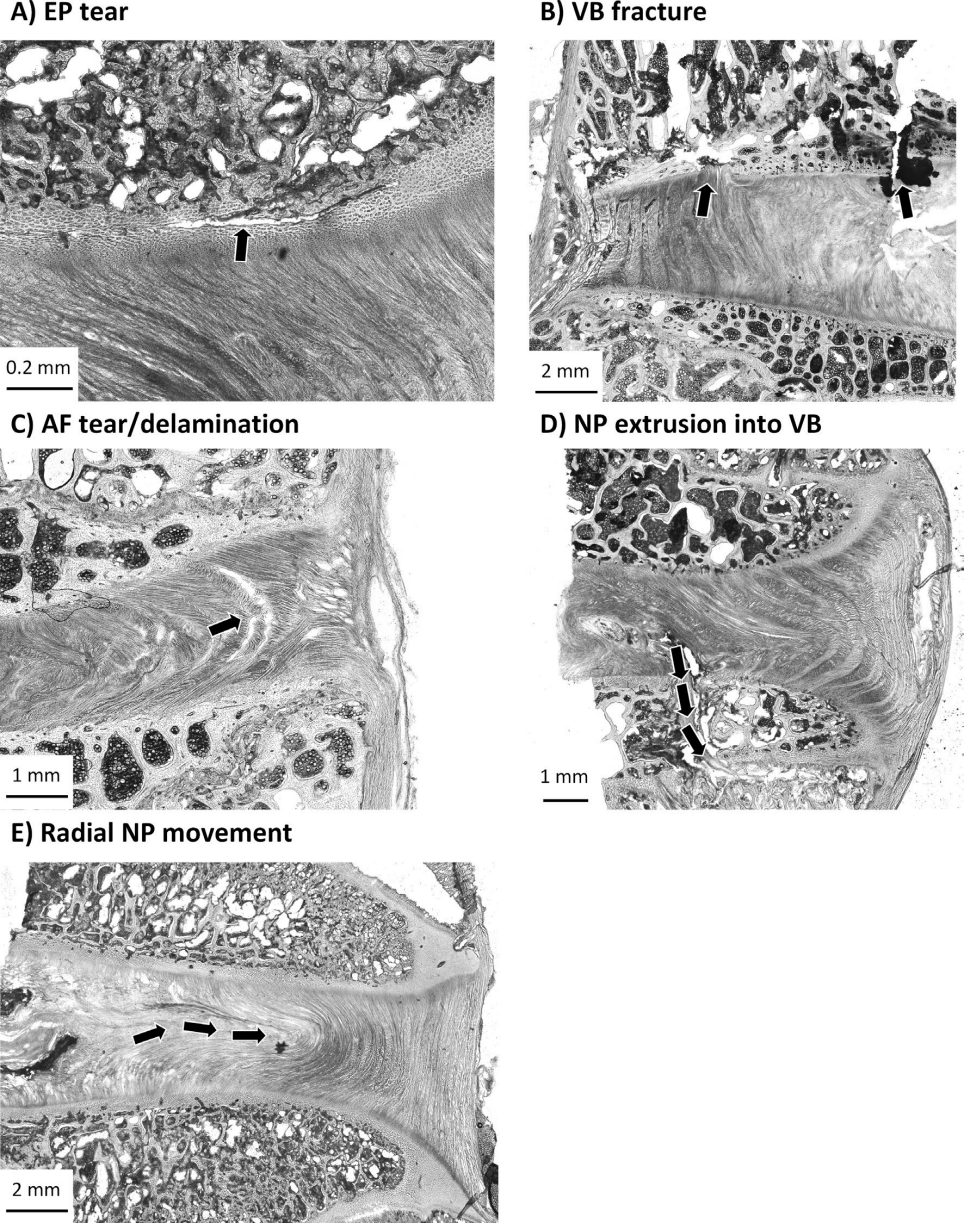
Microscopic images showing types of disc damage following shear‐compression loading. (A) EP tear (bovine lumbar); (B) VB fracture (ovine lumbar); (C) AF tear/delamination (ovine lumbar); (D) NP extrusion into VB (porcine cervical); (E) Radial NP movement (bovine lumbar). Arrows highlight examples of the described damage. Scale bars are included on all images.

Five structural damage features (Endplate (EP) tear; VB fracture; AF tear/delamination; NP extrusion into VB; radial NP movement) were defined to enable comparisons across species. All damage identified could be characterized as one of these categories. If the specific type of damage was found within the disc, it was recorded (as 1); otherwise, it was marked as not present (0). Example images and descriptions of these categories are presented in Figure 2. Damage was recorded by two independent researchers (BG and TS), and discrepancies (~10%) between findings were investigated until consensus was reached. Two control discs per species were prepared to assess the effects of fixation, decalcification, sectioning, and imaging. No notable artifacts from the microstructural imaging process were found. Representative control images for each species are provided in Appendix B.

### 2.8 Statistical Analyses

The peak forces and apparent pressures at failure between species were analyzed using a one‐way ANOVA to determine if these factors influenced failure mechanisms across species. Normality of the data was checked using the Shapiro–Wilk test, and homogeneity of variances was assessed using Levene's test. If significant differences were found from the ANOVA, a post hoc Tukey's HSD test was performed to identify which species differed.

The correlations between five external indicators (the four audio‐visual parameters and the load‐drop stop criterion) were assessed individually against each of the five observed microstructural damage categories. Due to the binary nature of the data, Fisher's Exact Test was used, with a Bonferroni‐corrected significance threshold of *p* < 0.002.

## 3 Results

**FIGURE 3:**
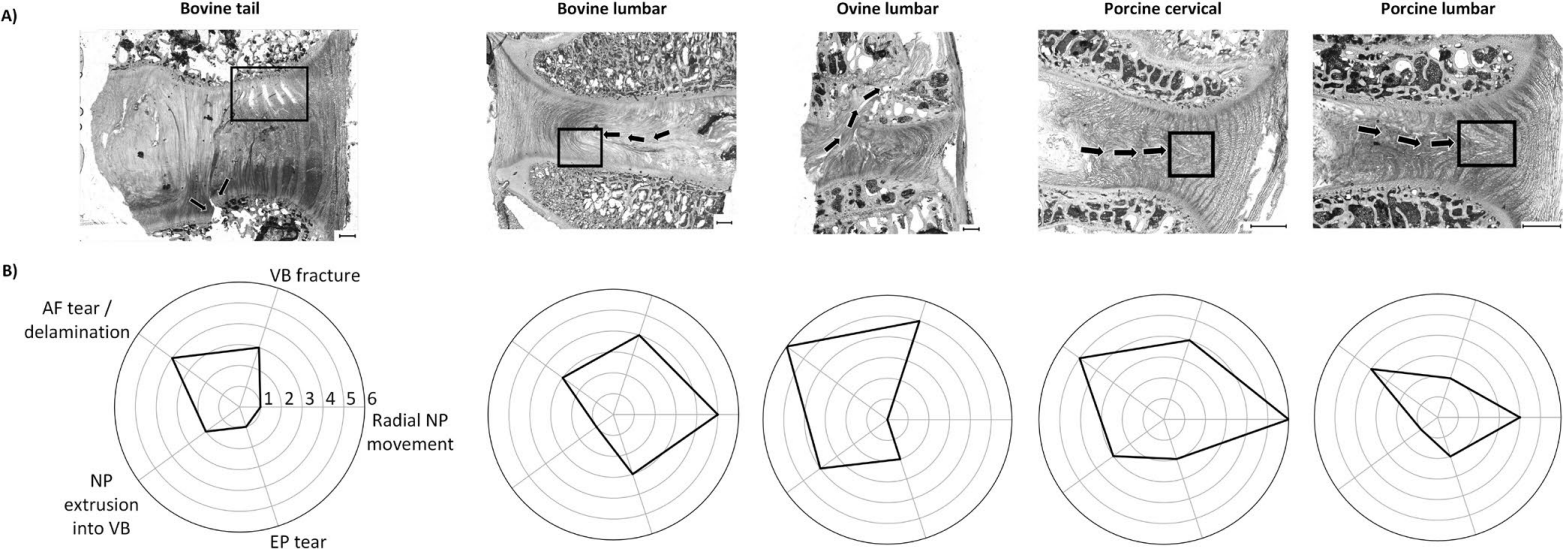
(A) Representative microstructural section highlighting typical damage patterns and failure types across specimens. Black arrows indicate material movement, whereas black boxes mark AF tears/delaminations. A 1 mm scale bar is included on each image. (B) Radar charts illustrating the frequency of observed damage types in each species. The first radar chart (Bovine tail) is labeled with the five microstructural damage categories, with each axis representing a specific damage type. The radius of each axis corresponds to the number of segments from (0 to 6) exhibiting the respective damage.

Eighteen (60%) discs failed due to the force drop, and the remaining 12 (40%) reached the displacement stop criterion (Table 2). Microscopic slide images of each disc are available in Appendix A. A representative section from each species and spinal region is presented, along with a radar plot summarizing the damage from all six species and regions (Figure 3). The 30 discs demonstrated instances of the five damage categories a total of 75 times. In 58% of these instances, damage was observed on both the left side and right side of the disc (56 on the left; 63 on the right).

**TABLE 2:** Disc characteristics and failure categories (audio‐visual; microstructural), grouped by species.

Animal model	Displacement (D) or load (L) stop criteria	Load at failure/kN	Audio recording		Video recording			Microscopic damage features				Geometric measurements	
			Small and continuous cracks	Loud and sudden snap	Fluid ejection	Posterior displacement of superior vertebra	EP tear	VB fracture	AF tear/delamination	Extrusion of NP into vertebra	Radial NP movement	Height/mm	Area/mm^2^
Bovine tail	L	8.69		✓		✓		✓				4.16	399
	D	8.50	✓				✓			✓		5.16	517
	L	5.99		✓				✓	✓		✓	5.21	478
	L	6.12	✓	✓	✓	✓			✓			5.89	591
	L	5.27	✓		✓				✓	✓		6.14	516
	D	4.93			✓			✓	✓			6.57	407
Total or average (SD)		6.58 (1.62)	3	3	3	2	1	3	4	2	1	5.52	483
Bovine lumbar	D	4.48					✓	✓			✓	3.96	700
	D	3.81	✓		✓		✓				✓	3.94	744
	D	4.75	✓		✓			✓	✓			3.30	736
	D	4.86	✓		✓			✓	✓		✓	3.75	764
	L	2.13	✓			✓			✓		✓	3.79	713
	L	2.33	✓			✓	✓	✓			✓	4.57	782
Total or average (SD)		3.73 (1.21)	5	0	3	2	3	4	3	1	5	3.89	741
Ovine lumbar	L	4.39		✓		✓		✓	✓	✓		2.87	306
	L	4.34	✓	✓			✓	✓	✓	✓		3.25	302
	L	5.05	✓	✓			✓	✓	✓	✓		3.47	351
	L	3.13	✓	✓		✓		✓	✓			2.96	275
	L	4.24	✓	✓	✓			✓	✓	✓		2.98	305
	D	3.95	✓						✓			2.85	335
Total or average (SD)		4.18 (0.63)	5	5	1	2	2	5	6	4	0	3.06	312
Porcine cervical	L	3.50	✓	✓		✓		✓	✓	✓	✓	3.22	507
	D	5.38	✓		✓				✓		✓	3.10	537
	D	0.95		✓	✓			✓	✓		✓	3.64	663
	L	1.42	✓		✓			✓		✓	✓	3.29	650
	L	4.13	✓	✓		✓	✓	✓	✓		✓	3.42	596
	L	3.63					✓		✓	✓	✓	3.45	627
Total or average (SD)		3.17 (1.68)	4	3	3	2	2	4	5	3	6	3.35	596
Porcine lumbar	L	2.57	✓		✓						✓	4.49	452
	D	3.35			✓		✓		✓		✓	3.56	450
	D	3.28									✓	3.37	430
	L	3.55	✓		✓				✓		✓	4.07	440
	L	3.20		✓	✓		✓	✓	✓			3.72	506
	D	3.91		✓	✓	✓		✓	✓	✓		3.41	460
Total or average (SD)		3.31 (0.44)	2	2	5	1	2	2	4	1	4	3.77	456

*Note:* Height and area measurements are color‐coded, with green indicating the largest discs, as these most closely resemble human lumbar disc dimensions (area ~1700 mm^2^; height ~9 mm [55]). Standard deviations are given for the average load at failure and average geometric measurements for each animal model.

Failure features varied between species, highlighting distinct structural responses to combined loading (Figure 3). In Figure 3, the area covered by each plot reflects the extent of structural damage observed, with larger areas indicating more extensive damage. Ovine lumbar segments showed the most extensive structural damage, with the greatest recorded damages for AF tear/delamination (6/6) and VB fracture records (5/6). Porcine cervical discs also exhibited substantial internal disruption, with consistently high damage records for radial NP movement (6/6) and AF tears (5/6). In contrast, bovine tail segments had relatively low structural damage overall, with only moderate VB fracture (3/6) and AF tearing (4/6). Bovine lumbar discs showed high radial NP movement (5/6) but minimal NP extrusion through the VB itself. EP tearing remained low across groups, but was slightly more frequent in bovine lumbar segments (3/6).

**FIGURE 4:**
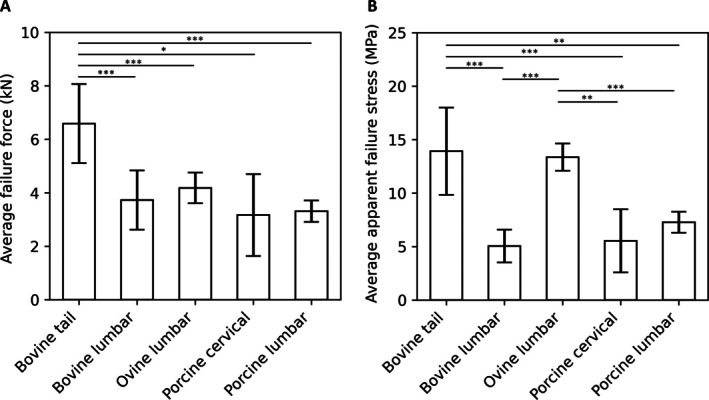
Comparison of peak failure forces (A) and corresponding apparent stresses (B) with mechanical testing terminated according to predefined failure criteria. The error bars are the standard deviations from the data. Asterisks denote statistically significant differences between groups (*p < 0.05, **p < 0.01, ***p < 0.001).

The peak forces and apparent stress at failure are plotted in Figure 4. Peak failure force was significantly higher in the bovine tail (6.59 ± 1.48 kN) than in any other species (*p* < 0.0177) and was approximately 60% higher than the other failure groups. When normalized to cross‐sectional area, the apparent stress at failure was, again, significantly higher in both the bovine tail (13.9 ± 4.1) MPa, (*p* < 0.002) and also the ovine lumbar (13.4 ± 1.3) MPa, (*p* < 0.005) relative to the other three species.

No significant associations were found between audio‐visual data and stop criteria or microstructural damage. However, loud, short snaps showed a near‐significant correlation with vertebral body fractures (*p* = 0.0024), which became significant (*p* = 0.0005) when bovine lumbar data (4/6 fractures, no loud snaps) were excluded, suggesting such snaps may indicate VB fractures in other species.

### 3.1 Variations Within Microscopic Damage Categories

**FIGURE 5:**
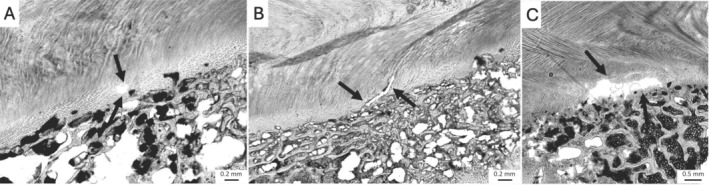
Examples of EP tears, as indicated by the arrows. (A) Hole in the EP itself, bovine lumbar; (B) tear along the fiber direction, bovine lumbar; (C) large horizontal tear, porcine lumbar.

**FIGURE 6:**
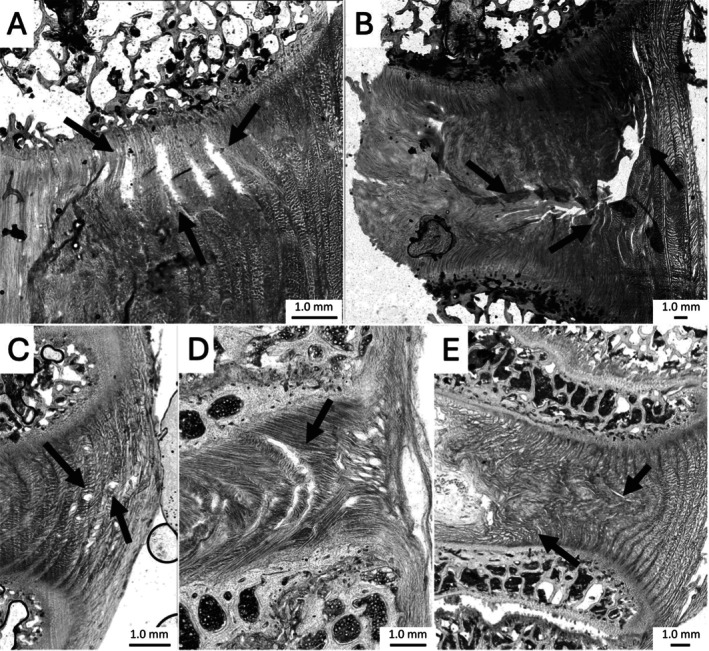
Examples of AF tears/delaminations, indicated by arrows. (A) Tears in alternating lamellae, bovine tail; (B) tear spanning several lamellae, bovine tail; (C) small perforations in the AF fibers, porcine lumbar; (D) tear of the interlamellar fibers, ovine lumbar; (E) tears aligned with the direction of NP material movement, porcine lumbar.

Microscopic damage morphologies varied within the binarized categories, especially in EP and AF tears. EP tears were seen to occur in three ways: small holes between the AF fibers and caudal EP (Figure 5A), fiber‐aligned tears with partial detachment of the EP (Figure 5B), and horizontal detachment of the EP from the VB (Figure 5C). AF tears/delaminations occurred in five distinct modes: alternating lamellar tears (Figure 6A), bridging failures across multiple lamellae (Figure 6B), smaller holes in the AF (Figure 6C), separation of lamellae due to damage in bridging fibers (Figure 6D), and damage along the direction of NP material movement (Figure 6E).

## 4 Discussion

This study investigated the microstructural failure modes of animal intervertebral discs under a standardized load to better understand their relevance to human disc herniation and to guide model selection in future research.

The observed failures across all discs could be broadly categorized into two generic failure modes: VB fracture‐driven failures and NP directional movement failures. VB fracture‐driven failures were typically associated with a loud snap and consistently met the load‐failure criterion. In contrast, NP directional movement failures were characterized by the NP protruding into the inner or mid‐AF fibers. These two failure patterns appear to align well with clinical classifications: EPJF‐type herniations typically involve vertebral fractures [11], whereas AF‐driven herniations require NP directional movement in most cases [56].

Under the applied loading regime, the likelihood of a particular failure mode was influenced by species and spinal region. Ovine lumbar discs predominantly exhibited VB fracture‐driven failures (84%), suggesting EPJF‐type herniations were more common. In contrast, porcine cervical (84%), bovine lumbar (84%), and porcine lumbar (66%) discs more frequently showed directional NP movement, consistent with AF‐driven herniations. The bovine tail discs fit into neither category of broad failure, remained relatively undamaged, and displayed minimal interaction between failures (e.g., while annular tears occurred in 67%, only 16% showed radial NP as a comorbidity).

The right‐hand side of the disc was expected to experience greater tensile and shear stresses due to the applied lateral bending [49, 50]. However, microstructural damage was not restricted to this region, with the same structural failure observed on both sides of the disc in 58% of cases. This similarity in damage distribution may be related to several factors, including: (i) inhomogeneous strain distributions within the disc during loading [55]; (ii) biological variations in disc anatomy, particularly in the AF [57], and (iii) the relatively small magnitude of the 5° transverse‐plane offset, which was likely insufficient to consistently bias damage toward the right side of the disc, especially given the biological variability of the discs.

### 4.1 Comparing Animal Models in Existing Literature

Although previous studies have focused on single animal models to examine disc herniation and failure mechanisms under applied loads [51], our study offers a unique comparison across multiple species. For instance, in our study, VB fractures were commonly observed in the ovine model, consistent with previous findings [9, 10], however, we did not observe any radial NP movement. This may be due to the posterior and lateral shear loading applied to our model. Some studies remedy this through incision of the annulus [17, 27, 36], though this approach may artificially reduce the likelihood of endplate junction failure (EPJF), limiting the physiological relevance of the model [11].

Whilst VB fractures have been frequently observed in ovine models [9, 10, 16, 23, 58, 59], porcine cervical [32, 33, 34, 35], porcine lumbar [60, 61], and bovine lumbar [36], this study is the first to quantify that ovine lumbar are the most likely to undergo VB fracture and that this occurs at a higher failure stress (~14 MPa) than many other species (bovine lumbar, porcine cervical, and porcine lumbar fail at ~4–8 MPa).

### 4.2 EPJF and AF‐Failure of the Disc

High compressive loads were required to induce failure in non‐degenerate segments, often resulting in damage to both the discs and adjacent VBs. Failure often occurred at the disc–VB interface and included VB fracturing, consistent with endplate junction failure [10, 14, 58]. Although this may deviate from disc‐specific damage, EPJF is a clinically relevant failure mode that reflects the mechanical integration of the disc–VB unit [11]. Similar patterns have been observed in previous studies using mechanical herniation loading protocols [14, 58].

The damage patterns observed in this study provide valuable insight into structural failure mechanisms that may contribute to, or precede, clinically relevant disc herniation. If the objective of a study is to isolate a specific failure mode, researchers must account for biological variability and be prepared to exclude specimens that do not exhibit the desired failure pattern.

### 4.3 Inter‐Species Differences in Failure Stress

Bovine lumbar discs showed the lowest failure stress (~5 MPa), whereas bovine tail and ovine lumbar discs had significantly higher values (~13 MPa), indicating greater load resistance (Figure 2). These differences likely reflect variations in bone mineral density (BMD), as vertebral fractures occurred in 60% of specimens, and BMD is a known predictor of fracture risk in humans [62, 63]. Published data show that vertebral BMD is higher in all tested species compared to humans (typically from 1.5 to 2.5× higher) except in juvenile bovine lumbar specimens, whose BMD may approach human levels due to age‐related immaturity [64, 65, 66, 67, 68]. Models with higher failure stresses, such as bovine tail or ovine lumbar, may be better suited for simulating acute failure or testing implants under supra‐physiological loads. Given that peak in vivo human disc pressures reach ~3 MPa [ 69, 70], a 4× safety factor supports their relevance for such applications.

### 4.4 Experimental Method Standardization

During standardization of the flexion angle, this study introduced unavoidable compromises in how closely the loading reflected the in vivo biomechanics of each spinal region. Anatomical and mechanical differences such as the high flexibility of the bovine tail [71] and the lower mobility of the ovine lumbar spine [42] mean that a uniform 7° flexion does not represent an equivalent physiological loading state across specimens. These differences may have influenced the resulting microstructural damage, which is a limitation of the study.

Variation in disc height across species introduced different strain rates, potentially affecting the internal pressure and failure behavior of the discs. However, the small differences in strain rates likely had minimal impact on disc stiffness [22] and hence, failure modes.

The load‐drop criterion was met in 83% of tests, indicating that the displacement criterion, which served only to prevent excessive compression, was set correctly. Although the bovine lumbar discs reached the displacement limit in 50% of cases, two of the discs failed at 2 mm of compression, considerably below the 5 mm threshold. This suggests a high variability in the failure displacement of bovine lumbar segments.

### 4.5 Estimated Microstructural Damage in Clinical Disc Herniation

**FIGURE 7:**
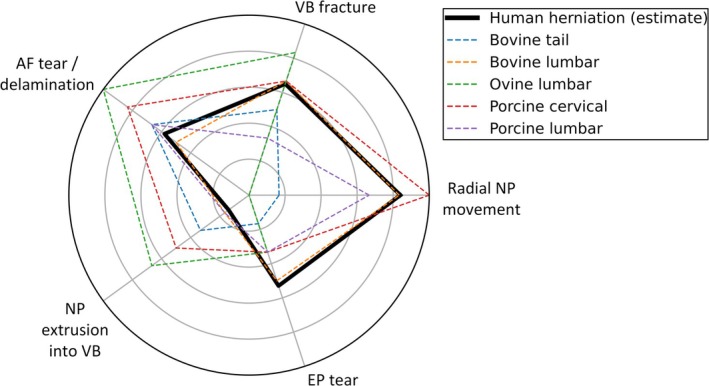
Radar chart depicting an estimation of structural damage associated with lumbar disc herniation in vivo (represented by the thick black line) and animal model herniation (represented by colored dashed lines). The radius of each point reflects the percentage of herniations exhibiting, or estimated to exhibit that type of microstructural damage, with the centre of the circle representing 0%, and the outer edge 100% of herniations. The human radar plot was based on clinical data, including histopathology and imaging studies [11, 56, 72].

The radar chart for human disc herniation (Figure 7) was based on histological and imaging studies (Table 3). Comparison with animal models showed that bovine lumbar discs display a similar damage profile, suggesting analogous failure mechanisms, such as AF rupture, NP extrusion, and VB fracture, though without NP extrusion through the VB. This observation supports the use of bovine lumbar models for studying herniation progression.

However, of the animal models, the bovine lumbar model did not exhibit the greatest extent of damage overall; both the porcine cervical and ovine lumbar models showed greater counts of microstructural failure (Figure 3). This suggests that while bovine lumbar discs may replicate certain key features of human herniation, other models may better represent broader or more advanced damage states, depending on the specific pathology or injury mechanism being studied.

This approach, as a comparison to human disc herniation, has several limitations. The chart represents an estimation of microstructural damage, reflecting common patterns observed in human specimens but without specifying which features are most likely to co‐occur. These patterns may vary depending on distinct clinical pathways, such as AF failure or EPJF [11, 12]. Additionally, the radar plot indicates only the presence of damage, without capturing its extent, chronology, or mechanical implications. To address these limitations, future studies should aim to quantify the progression of microstructural damage in human discs leading to herniation (Table 3).

**TABLE 3:** Estimations for the microstructural damages within a human herniation made using inferences from clinical data.

Damage type	Estimated percentage	Reason
VB fracture	65%	EPJF is implicated in 65% of patients. VB fractures are found in all cases ranging from small free‐fragments (Type 1A); to full endplate avulsions (Type 1D) [11].
NP extrusion into the VB	14%	Bony‐avulsions have occurred within the vertebra in 14% of patients; NP may then extrude into the vertebra; and herniate the disc [11].
Radial NP movement	86%	In all cases where NP has not extruded through the VB (row above: 86%), it has been assumed to have traveled radially through the disc leading to herniation [11]. NP was also found in 98% of excised tissue; demonstrating that NP is found to move radially in nearly all herniation cases [56, 72].
AF tears	58%	Annulus fibrosus (AF) tears were present in 58% of degenerate discs [56]. This value has been assumed for a herniated disc, which are usually found to be degenerate [73, 74].
EP tears	53%	EP tearing, inferred from cartilaginous EP removal during surgery, is observed in 53% of herniated discs it [56].

### 4.6 Recommendation for Animal Model Choice

**FIGURE 8:**
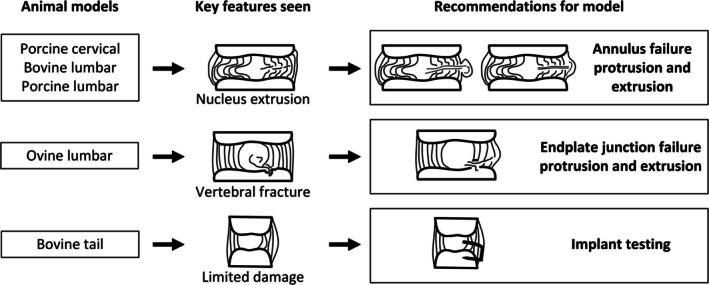
Recommendations for each animal model based on the microstructural damage recorded and anatomical measurements of the discs.

Three categories of recommendations were established based on this study (Figure 8): bovine lumbar, porcine lumbar, and porcine cervical discs were identified as the models most suitable for studying AF failure protrusions and extrusions. Ovine lumbar discs were best suited for modeling endplate junction failure due to their high rate of vertebral fractures (86%) and lack of radial NP movement (through the annulus) (0%). Bovine tail discs, with their larger size and greater disc height and limited damage profile, are recommended for implant studies where anatomical dimensions may be a primary consideration.

To refine these recommendations, additional observations were made regarding the microstructural damage patterns in each model. The bovine lumbar disc radar chart most closely resembled human clinical herniation based on estimated microstructural damage (Figure 7). However, we suggest that porcine cervical discs may be the best‐suited model for studying AF failure studies due to their higher damage rates than both porcine lumbar (in 4/5 categories) and bovine lumbar discs (in 3/5 categories). This higher failure rate across multiple categories suggests that porcine cervical discs more reliably reproduce key human herniation features, potentially requiring fewer discs per study.

These recommendations are caveated by the fact that the results were derived from a single, standardized load, which may not generalize to all loading conditions. Additionally, the removal of facet joints, whereas necessary to isolate disc loading, resulted in less physiological movement during testing. It is therefore suggested that future studies using these models also remove facet joints to maintain consistency. Despite these limitations, all models can offer valuable insights into herniation, and continued research will help refine their applicability to different study contexts.

### 4.7 Cross‐Study Comparisons

Our study enhances understanding of herniation in animal and human discs, facilitating comparisons between prior research. Lundin et al. [61] and Wade et al. [9] (neutral + high rate group) conducted axial compression tests on porcine and ovine lumbar discs, respectively, applying loads for ~5 s [9, 61]. Failure mechanisms varied significantly: Lundin et al. observed VB fractures in 50% of tests; AF tearing in 33%; and NP radial movement in 84% of discs. In contrast, our data suggest that ovine lumbar discs would exhibit no radial movement (0%), a higher VB fracture rate (84%), and similar or increased AF tearing (100%). Similarly, Wade et al. reported 100% VB fractures, no herniations (indicating a lack of radial NP movement (~0%)), and didn't report AF tearing. These findings demonstrate how this study aids in interpreting results across animal models, even under differing loading conditions. However, it should be noted that hydration maintenance protocols varied across these studies (e.g., hydrating specimens via water bath immersion [9] and placing specimens in plastic bags [61]), which likely influenced the disc's internal pressurization mechanics. This limits direct comparability of microstructural failure modes, even when loading parameters are similar.

### 4.8 Limitations of the Study

Microstructural imaging of whole discs presents challenges. The 50 μm section thickness makes it challenging to keep the entire microstructure in‐plane and fully resolved. In some cases, features such as fiber tears or bone fractures may become more apparent during sectioning. Future studies should utilize both micro‐ and macro‐scale imaging to better characterize disc condition prior to sectioning.

The use of skeletally immature spines may limit direct comparison to human herniated discs, which are most common in individuals between the ages of 30–50 [3, 75]. This model was chosen to represent healthy, non‐degenerate tissue for modeling early‐stage mechanical injury. However, the skeletal immaturity of these specimens may lead to a higher incidence of vertebral body and endplate failures [76]. Therefore, specimen maturity should be carefully considered when interpreting failure properties.

The moderate loading rate chosen of 40 mm min^−1^ may not fully replicate physiological injury speeds, and different loading rates may impact the types of damage observed [10]. Additionally, further refining the termination criteria to be specimen specific could improve the comparability of the damage observed across discs. Tailoring the criteria to account for variations in disc properties or damage progression may lead to more consistent and accurate interpretations of injury mechanisms.

Although group sizes (*n* = 6 per species) enabled consistent comparisons across models, they limited the statistical power to detect subtle interspecies differences. Nevertheless, the differences in observed failure modes and microstructural findings support the validity of the approach and highlight the potential of this model for further comparative investigations.

## 5 Conclusions

This study examined microstructural damages within a herniation model across species and spinal regions using a standardized mechanical load. Based on the observed microscopic damage and estimated damage in clinically herniated discs, porcine cervical, bovine lumbar, and porcine lumbar discs were the most effective models for extrusion or protrusion‐type herniations, characterized by radial NP movement. However, porcine cervical discs showed the highest damage rates, making them the preferred model due to their greater consistency in replicating these herniation features. Ovine lumbar discs were particularly suited for modeling EPJF due to high rates of vertebral fracture. Bovine tail discs demonstrated limited amounts of damage, and due to their large disc height and area, were recommended for implant studies.

## Conflicts of Interest

The authors declare no conflicts of interest.

## Figures and Tables

**FIGURE A1:**
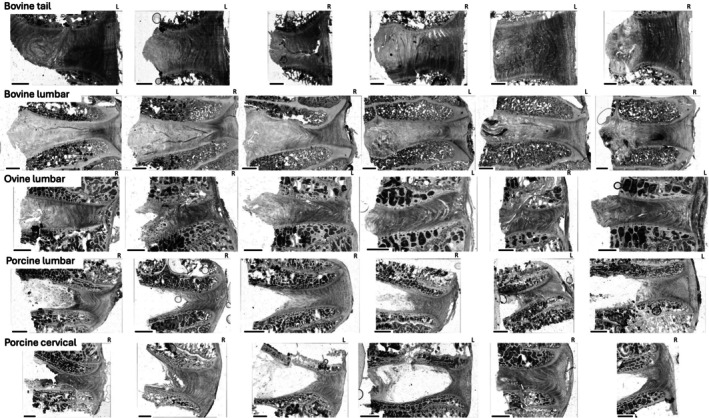
Microscopic section images from each mechanically tested segment, showing either the left (L) or right (R) side of the disc. The slice selected best represents the damage observed in that specimen. Images are shown in the same order as the segments listed in Table 2. Left (L) and right (R) sides of the disc are indicated on the images. The scale bar in each image represents 2 mm.

**FIGURE B1:**
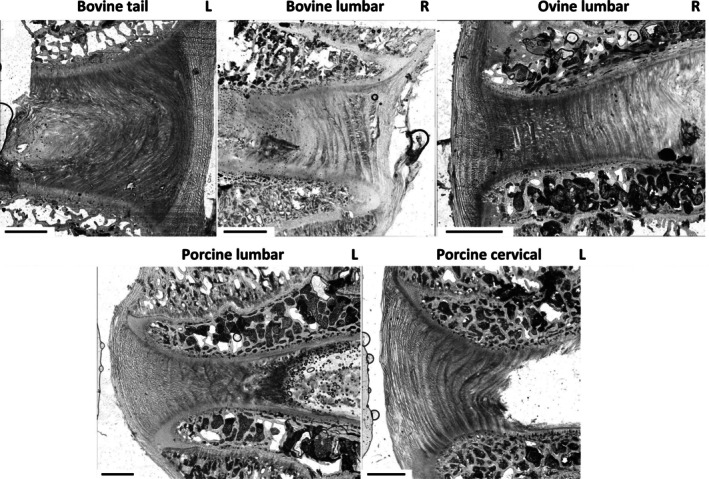
Microscopic section images of five control intervertebral discs, showing either the left (L) and right (R) sides of each disc segment. The scale bar in each image represents 2 mm.

## Data Availability

The data that support the findings of this study are available from the corresponding author upon reasonable request.

## Appendix A Microscopic Sections From All Tested Discs

See (Figure A1).

## Appendix B Microscopic Sections From Control Discs

See (Figure B1).
